# Complete chloroplast genome of the wild-type *Monimopetalum chinense* (Celastraceae)

**DOI:** 10.1080/23802359.2019.1624207

**Published:** 2019-07-12

**Authors:** Jian Pan, Zhen Fang, Dong-Ming Ji, Tao-Hong Gu, Jia-Shou Cheng, Xiao-Ming Yu, Shuai Li, Fu-Rong Ye

**Affiliations:** a College of Life and Environment Sciences, Huangshan University, Huangshan, P. R. China;; b Zhawan Forest Industry Breeding Farm of Qimen County, Huangshan, P. R. China;; c Forestry Bureau of Qimen County, Huangshan, P. R. China

**Keywords:** *Monimopetalum chinense*, wild-type, chloroplast genome, phylogeny

## Abstract

*Monimopetalum chinense* (Celastraceae), belonging to the monotypic genus, is an endangered species endemic to eastern China. The complete chloroplast (cp) genome sequence of *M*. *chinense* was determined using next-generation sequencing technology. Total length of the cp genome is 159,208 bp, which contained a large single-copy (LSC) of 88,024 bp in length and a small single copy (SSC) of 18,662 bp, separated by two inverted repeats (IRs) of 26,261 bp. The cp DNA is structured with 133 genes, comprising 88 protein-coding genes, 37 tRNA genes, and eight rRNA genes. Phylogenetic analysis suggested that *Monimopetalum* is closely related to the genus *Catha* and *Euonymus* with in the Celastraceae.

*Monimopetalum chinense* belonging to the monotypic genus, the only member of the genus *Monimopetalum* (Celastraceae), is an endangered species endemic to eastern China. This species has a small geographic range that limited mountain area of the middle and lower reaches of Yangtze river. It only distributed from south of Anhui to north of Jiangxi and southeast of Hubei. In recent decades, the natural populations have been undergone a rapid demographic decline, mainly due to habitat destruction. Its natural populations are small and isolated, and it is classified as an endangered species in the Chinese Plant Red Book (Fu 1992). Previously some researches concerning *M*. *chinense* focused on ecological characters and breeding techniques (Xie and Tan 1998; Xie and Wen 1999; Xie and Zhang 1999; Liu and Yao 2016; Sun et al. 2017) and the genetic diversity and population structures of *M*. *chinense* were reported (Xie et al. 2005; Li et al. 2009; Li et al. 2011). This study, complete cp genome of the wild-type *M*. *chinense* collected from Qimen County, Anhui Province was determined and described in order to provide basic genetic information about this endangered species.

Fresh leaves of *M*. *chinense* were sampled from the Qimen County, Anhui Province, China, the type locality of this species. It was preserved and deposited in the Museum of Huangshan University (Voucher number: HSP2018002). The cp genome sequence of *M*. *chinense* was determined using next-generation sequencing technology (Illumina Hiseq 2500) at Shanghai Majorbio Bio-pharm Technology Co., Ltd (Shanghai, China). Genome sequences were picked out and assembled by the software SOAPdenovo v2.04 (Luo et al. 2012). The cp genome of *M*. *chinense* (Genbank accession number MK450440) showed a typical structure with a length of 159,208 bp, which contained a large single-copy (LSC), a small single copy (SSC) and two inverted repeats (IRs). The cp DNA is structured with 133 genes, comprising 88 protein-coding genes, 37 tRNA genes, and eight rRNA genes. The overall base composition of the entire genome is as follows: G (19.0%), C (18.4%), A (30.90%), and T (31.7%), which the percentage of A + T is 62.6%.

The large single-copy (LSC) is 88,024 bp in length, including 61 protein-coding genes and 21 tRNA genes, the small single copy (SSC) is 18, 662 bp containing 11 protein-coding genes (ndhF, rpl32, ccsA, ndhD, psaC, ndhE, ndhG, ndhI, ndhA, ndhH, and rps15), one tRNA gene (tRNA-Leu), and the two inverted repeats (IRs) are 26,261 bp, including eight coding genes (rpl2, rpl23, ycf2, ndhB, rps7, rps12, ycf15, and ycf1), six tRNA genes (tRNA-Leu, tRNA-Val, tRNA-Ile, tRNA-Ala, tRNA-Arg, and tRNA-Asn), and four rRNA genes (4.5s rRNA, 5s rRNA, 16s rRNA, and 23s rRNA), respectively. The gene ycf1 is across the SSC and IR.

The whole cp genome sequence of the *M*. *chinense* determined in this study and together with other 10 closely related species sequences from GeneBank to perform phylogenetic analysis. A maximum-likelihood (ML) tree was constructed based on the dataset using the online tool RAxML (Kozlov et al. 2018). Phylogenetic analysis result suggested that *Monimopetalum* is closely related to the genus *Catha* and *Euonymus* with in the Celastraceae (Figure 1).

**Figure 1.**
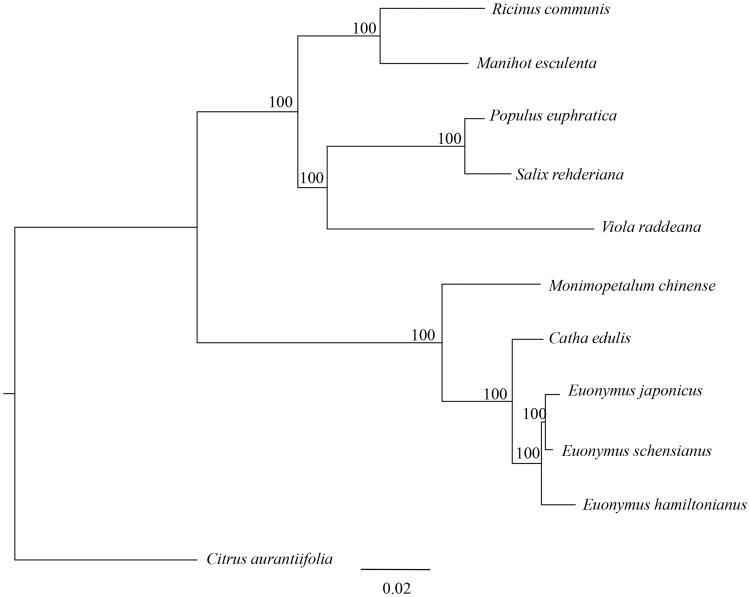
The phylogenetic tree (ML) was constructed based on the dataset of whole chloroplast genome. The numbers above the branch meant bootstrap value. The analyzed species and corresponding NCBI accession number as follows: *Ricinus communis* (JF937588), *Manihot esculenta* (EU117376), *Populus euphratica* (KJ624919), *Salix rehderiana* (MG262367), *Viola raddeana* (MH229818), *Monimopetalum chinense* (MK450440), *Catha edulis* (KT861471), *Euonymus japonicus* (KP189362), *Euonymus schensianus* (KY511610), *Euonymus hamiltonianus* (KY926695), and C*itrus aurantiifolia* (KJ865401).
